# Deep cfDNA fragment end profiling enables cancer detection

**DOI:** 10.1186/s12943-021-01491-8

**Published:** 2022-01-21

**Authors:** Yulia V. Zhitnyuk, Anastasia P. Koval, Aleksandr A. Alferov, Yanina A. Shtykova, Ilgar Z. Mamedov, Nikolay E. Kushlinskii, Dmitriy M. Chudakov, Dmitry S. Shcherbo

**Affiliations:** 1 Institute of Translational Medicine, Pirogov Russian National Research Medical University, 1 Ostrovityanova str, Moscow, Russia 117997; 2 Laboratory of Clinical Biochemistry, N.N. Blokhin National Medical Research Center of Oncology, 23 Kashirskoye Highway, Moscow, Russia 115478; 3 Federal Center for Brain and Neurotechnology, 1/10 Ostrovityanova str, Moscow, Russia 117513; 4 Department of Genomics of Adaptive Immunity, Shemyakin-Ovchinnikov Institute of Bioorganic Chemistry, 16/10 Miklukho-Maklaya str, Moscow, Russia 117997; 5 Dmitry Rogachev National Medical and Research Center of Pediatric Hematology, Oncology and Immunology, 1 Samory Mashela str, Moscow, Russia 117997

**Keywords:** cfDNA, Fragmentomics, cfDNA-FEP, Liquid biopsy, Early cancer detection, Renal cancer, Colorectal cancer

## Background

The number of cancer cases is expected to increase by 40% in 20 years and reach nearly 30 million new cases per year in 2040 [1]. Therefore, it is of utmost importance to get a grip on cancer prevention and early diagnosis. Colorectal cancer (CRC) is the third most commonly diagnosed and the second most deadly cancer worldwide [1]. Because it begins insidiously, 20% of CRC cases are not discovered until cancer has already outgrown the colon [2]. Detecting tumors at an early stage represents a major opportunity to reduce CRC morbidity and mortality and improve patient prognosis. Renal cell carcinoma is the ninth most common cancer worldwide, with an increasing incidence due to growing obesity rates, smoking and alcohol consumption. In most cases, renal cell carcinoma is diagnosed incidentally on imaging, and rarely presents with either classic symptoms such as hematuria and flank mass or paraneoplastic syndromes or varicocele in men [3]. 35% of renal cell carcinoma cases are detected at the metastatic stage, and no current screening test is available.

Cell-free DNA (cfDNA) found in the bloodstream is primarily a byproduct of cell death in both normal and cancer cells [4]. Circulating DNA fragments are mainly short molecules with an average length of mononucleosome size that tend to be more fragmented in internucleosomal linkers and open chromatin regions. This leads to a biased, non-random fragmentation pattern [5]. Moreover, tumor-derived DNA fragments (ctDNA) tend to be shorter than the non-tumor cell-derived fraction, and constantly accumulating evidence suggests that cfDNA fragmentation may serve as a cancer biomarker at the whole-genome level [6, 7]. Some studies argue the presence of specific genomic regions with preferential tissue-specific or tumor-specific cfDNA fragmentation [8]. Recently, several groups have thoughtfully characterized open chromatin landscapes in human cancer [9, 10], allowing further extrapolations to the cfDNA fragmentation footprints [11]. Here, we focus on targeted high-resolution profiling of cancer-specific open-chromatin regions in cfDNA from the blood of healthy individuals and patients with colorectal and renal cancers. We demonstrate that the proposed approach can facilitate cancer detection.

## Results

### Targeted fragment end profiling in cfDNA

**Fig. 1:**
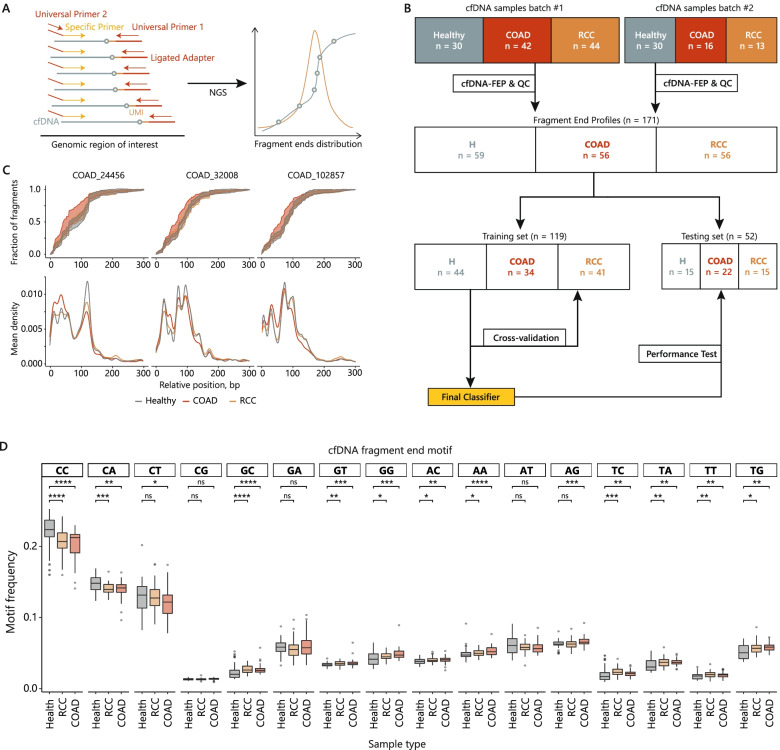
cfDNA-FEP overview and study design. **A** Schematic representation of cfDNA-FEP. Targeted amplification is done with a single specific primer for each target region and preserves the original fragment ends. Subsequent high-coverage NGS reveals the distribution of cfDNA fragment ends and sequence motifs. **B** Experiment design. The study cohort (*n* = 175) was split into two batches. Exploratory data analysis and feature preparation were done on the full dataset after quality control. Model tuning was done on the training subset, followed by a performance evaluation on the separate testing subset. **C** Fragment end profiles for stage IV cancer samples and healthy controls in 3 COAD-specific regions are visualized as empirical cumulative distribution functions (standard deviation range, top panel) and densities (bottom panel). **D.** Frequencies of cfDNA dinucleotide end motifs in healthy (*n* = 59), RCC (*n* = 56) and COAD (*n* = 56) samples. Significance determined with Wilcoxon test is indicated by asterisks (**** - *p* < 0.0001; *** - *p* < 0.001; ** - *p* < 0.01, * - *p* < 0.05, ns - not significant)

To design an assay capable of capturing cfDNA fragment end profile shifts in cancer, we examined the available ATAC-seq datasets of 410 tumor samples from The Cancer Genome Atlas (TCGA). These data characterize chromatin accessibility in 23 cancer types, including colon adenocarcinoma (COAD) and renal cell carcinoma (RCC) with a peak resolution of 500 bp [10]. Of these, we selected 48 COAD-specific and 48 RCC-specific peaks based on their normalized scores and specificity for the corresponding cancer type (Supplementary Fig. 1). To accurately analyze cfDNA fragment end profiles (cfDNA-FEP) in genomic regions of interest, we used a modified anchored multiplex PCR approach followed by NGS [12]. Briefly, ligation of the universal adapter to cfDNA is followed by primer extension from the target primer pool such that the resulting products contain universal adapter sequences at the 3′-ends. The ligated adapters contain unique molecular identifiers (UMIs) to effectively remove PCR duplicates during downstream analysis and converge read counts to the number of original cfDNA molecules. Subsequent amplification is performed with universal primers to reduce PCR biases. This scheme allows for targeted amplification while preserving information about the original end coordinates of the cfDNA fragments (Fig. 1A). The distributions of relative end positions reflect cfDNA fragmentation profiles specific to each target region. We hypothesized that the cfDNA end profile pattern in open-chromatin regions might differ between healthy individuals and cancer patients.

### cfDNA-FEP on a clinical cohort

A cohort of 175 individuals with histologically confirmed CRC (*n* = 58) and RCC (*n* = 57), as well as age-matched healthy individuals (*n* = 60), was divided into two batches (*n* = 116 and *n* = 59) that were processed individually to account for potential batch effects (Fig. 1B). After library preparation and paired-end next-generation sequencing, we performed UMI-clustering to remove PCR duplicates and then aligned clustered reads to the reference genome. To generate end profiles, we retrieved only proper pairs where the second reads overlapped the target primer binding sites. The relative start positions of the corresponding first reads represent the end profile of cfDNA molecules for each region (Fig. 1C, top). We examined the densities of the distributions in each target region and found that, in at least some regions, the average density at the peaks differed between cancer and control groups (Fig. 1C, bottom). These peaks in density denote sites where cfDNA is predominantly cleaved and may vary due to nucleosome repositioning, change in chromatin state, or aberrant nuclease activity associated with pathology [13]. To build a classifier, we selected the most prominent peaks in the range of 0–99 bp (Peak1) and 100–300 bp (Peak2) within each target region (Supplementary Fig. 2). The normalized log2 ratio of the densities in Peak1 and Peak2 served as a single-value metric of the fragmentation profile for each target region, or the fragmentation score (FS). We further noted that dinucleotides at the ends of the cfDNA fragments were not evenly distributed, with CC being the most frequent motif (Fig. 1D). This is consistent with the previous reports on hepatocellular carcinoma and may be a consequence of aberrant nuclease activity in cancer [14]. Therefore, we used the frequencies of sequence motifs along with FS values as predictors for the cfDNA-FEP model.

### Patient samples classification

**Fig. 2:**
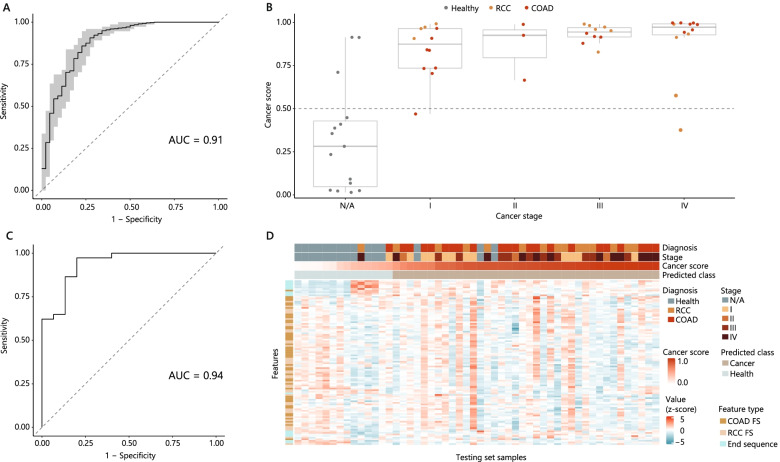
cfDNA-FEP performance. **A** ROC curve for the training set generated with 10-times 10-fold cross-validation (AUC = 0.91). Mean (solid line) and range (shaded area) are plotted. The dashed line denotes the theoretical performance of a random classifier. **B** Cancer scores predicted by the cfDNA-FEP classifier on the test set for COAD, RCC, and healthy samples stratified by clinical stage. A decision cutoff of 0.5 is denoted as a dashed line. **C** ROC curve for the testing set (AUC = 0.94). The dashed line denotes the theoretical performance of a random classifier. **D** The heatmap of fragmentomic features and characteristics of the test set samples (*n* = 52). cfDNA-FEP classification results are shown as cancer score and predicted class (cancer or health)

We trained support vector machine classifiers on a dedicated training dataset to select the best-performing model based on the area under the ROC curve. The final classifier was able to distinguish cancer and healthy samples on the training dataset (10 times 10-fold cross-validation) with a mean AUC = 0.91 (sd = 0.09, *n* = 100) (Fig. 2A) and on the unseen test dataset with an AUC = 0.94 and an accuracy of 0.9 (Fig. 2C). The cfDNA-FEP classifier generates a cancer score for each cfDNA sample. This metric reflects the probability that the cfDNA sample is from a patient with cancer (Fig. 2B). For samples from the test dataset, we observed only a slight decrease in classifier performance for early-stage (I, II) cancer (AUC = 0.91, accuracy 0.87) compared with late-stage (III, IV) disease (AUC = 0.96, accuracy 0.89). The median cancer score for healthy and stage I cancer samples was 0.275 (sd = 0.294, *n* = 15), and 0.831 (sd = 0.162, *n* = 12), respectively, making a classification of even early-stage cancers feasible with the decision cutoff of 0.5 (Fig. 2B). Stage IV cancer samples (*n* = 12) were labeled with a median cancer score of 0.955, sd = 0.205. The cfDNA-FEP was able to detect both cancer types in the test set with similar performance: AUC for RCC and COAD test set samples was 0.94.

## Discussion

Fragmentomic cfDNA features can be considered as independent analytes or additional biomarker layers in liquid biopsies. Several studies have demonstrated the utility of fragmentomic markers for cancer detection using whole-genome sequencing [8, 15, 16]. However, there are few reports of targeted assays [17] that are potentially more effective in the clinical setting due to their lower sequencing requirements and ability to complement existing approaches. Detection of cfDNA end profiles does not require additional treatments and can be combined with other NGS assays (the detection of cytosine methylation changes or somatic mutations). Moreover, current experimental evidence of cfDNA fragmentomic-based tumor detection is enriched with cancer types that are believed to shed more ctDNA into the bloodstream (e.g., liver, colorectal, lung, and breast), while fewer reports of successful detection of low-shedding cancers, including renal, are available [16]. In this report, we show that deep targeted profiling of cfDNA ends distributions and sequence motifs can reveal the presence of early-stage colorectal and renal cancers with an AUC = 0.94. The limitation of our study design is the lack of a group with benign pathological lesions in the colon and kidneys, so our results do not demonstrate the ability of the approach to distinguish cancer from other forms of tissue damage. Another direction for improvement would be a wider screening for additional targets derived from sources other than ATAC-seq data, such as regions of stable nucleosome repositioning specific to cancer cells or tumor-specific transcription factor binding sites.

## Conclusion

Our results show that deep profiling of cfDNA fragment ends can facilitate the detection of colorectal and renal cancers. We believe that cfDNA-FEP can be further extended to non-invasively detect more cancer types with higher precision.

## Supplementary Information

**Additional file 1. **Supplementary Methods.

**Additional file 2: Supplementary Fig 1.** ATAC-seq signal in RCC (KIRP and KIRC) and COAD-specific open-chromatin regions (rows) analyzed in this study shown for the samples (columns) from the TCGA cohort. Data from [10].

**Additional file 3: Supplementary Fig 2.** Densities of fragment end distributions in all target regions analyzed in this study plotted for COAD, RCC, and healthy cfDNA samples. Black vertical lines represent positions of Peak1 and Peak2.

**Additional file 4: Supplementary Fig 3.** Demographic and clinical characteristics of the cohort. **A-C.** Age and sex distribution across clinical groups (A), batches (B), and train/test split (C). **D.** The cfDNA yields across clinical groups and cancer stages. **E, F**. Stage and diagnosis composition of the batches (E), training and test sets (F).

**Additional file 5: Supplementary Table S1.** Cohort Characteristics.

**Additional file 6: Supplementary Table S2.** List of Used Oligonucleotides.

## Data Availability

Code and cfDNA fragment end positions in target regions for all samples are available from the GitHub repository https://github.com/dshcherbo/cfDNA-FEP. Sensitive patient-derived cfDNA sequencing data is available from the corresponding author upon reasonable request.

## Abbreviations

ATAC-seq: Assay for transposase-accessible chromatin using sequencing
AUC: Area under the curve
cfDNA: Cell-free DNA
cfDNA-FEP: Cell-free DNA fragment end profiling
COAD: Colon adenocarcinoma
CRC: Colorectal cancer
ctDNA: Circulating tumor DNA
FIT: Fecal immunohistochemical test
FS: Fragmentation score
KIRC: Kidney renal clear cell carcinoma.
KIRP: Kidney renal papillary cell carcinoma
NGS: Next-generation sequencing
RCC: Renal cell carcinoma
ROC: Receiver operating characteristic
UMI: Unique molecular identifier
