# Supplementary material for: Deep cfDNA fragment end profiling enables cancer detection
Source: Mol Cancer. 2022 Jan 21;21:26. doi: 10.1186/s12943-021-01491-8 (PMC8780681; doi:10.1186/s12943-021-01491-8)
Supplement: Supplementary file 1 — Additional file 1. Supplementary Methods. [file 12943_2021_1491_MOESM1_ESM.docx]

# Supplementary Methods

## Patient characteristics

Blood samples were collected prospectively from previously untreated individuals with CRC or RCC and healthy donors. A cohort of 175 individuals included 3 groups stratified by cancer type and balanced by age and sex (Supplementary Table 1, Supplementary Figure 3). Examination and treatment of patients with CRC and RCC were carried out at the N.N. Blokhin Medical Research Center of Oncology. In all patients, the clinical and radiological diagnoses were confirmed with the morphological examination of the tumor according to the WHO Classification. Adenocarcinoma of varying degrees of differentiation was revealed in all patients with CRC, clear cell carcinoma predominated in the group of patients with RCC. The samples from apparently healthy subjects were collected at the Federal Center for Brain and Neurotechnology.

## Blood and cfDNA processing

For cell-free DNA extraction 9 ml of blood was collected in PAXgene Blood ccfDNA Tubes (QIAGEN). Plasma was separated according to the manufacturer's recommendations immediately after collection, then cfDNA samples were extracted from the whole obtained plasma volume according to the manufacturer's protocol using the QIAamp MinElute ccfDNA Midi Kit (QIAGEN). Cell-free DNA was eluted into 30 μL of ultrapure water. The cfDNA samples were quantified with the Qubit dsDNA HS Assay Kit (Thermo Fisher Scientific) (Supplementary Table 1) and stored at -20°C prior to library preparation.

## Library preparation and NGS

Anchored multiplex PCR approach [1] was modified for the study. The equivalent portion (25 from 30 μL) of each cfDNA sample was used for library preparation. cfDNA was processed using NEBNext Ultra II End Repair/dA-Tailing Module (NEB) according to the manufacturer's protocol. Then the ligation of the UMI-containing universal adapter was performed using 5U of T4 DNA ligase (Thermo Fisher Scientific) for 30 min at RT. The next step was the primer extension with target primer pool with the following program: 3 min at 95°C, 2 min at 98°C, 10 cycles of 15 sec at 98°C, 5 min at 64°C, final elongation 5 min at 72°C. Target DNA fragments were amplified with universal primers using the following program: 3 min at 95°C, 2 min at 98°C, 24 cycles of 15 sec at 98°C, 2 min at 60°C, final elongation 5 min at 72°C. Target primer sequences are listed in Supplementary Table 2. The final library pool was sequenced on the Illumina NextSeq 500 instrument with 2x100nt paired reads. The first sample batch pool was sequenced on two Mid Output runs, the second batch pool was sequenced on a single Mid Output run.

## Targeted cfDNA fragment end profiling

Adapter sequences were removed from raw reads with cutadapt v2.7 [2]. UMI clustering to remove PCR duplicates was done with MiNNN v10.1. Processed reads were aligned to the reference human genome hg38 with bowtie2 v2.3.5.1 [3]. From the alignments, we extracted read pairs where the second reads aligned to binding sites of the target primers with mapping quality 20 or higher. Next, we retrieved the coordinates of the starts of the first reads relative to primer binding sites and the first 2 nucleotides of the reads. We limited the analysis to the region 0-300 bp. Quality control included a target read count filter (samples with target read counts < 10000 were filtered out). We further analyzed densities of the obtained relative coordinates distributions for each primer and found two maxima: in range 0-99 bp and 100-300 bp (Supplementary Figure 2). After that, we computed areas under the density curves within ±5 bp from both maxima and found the ratio of these two values for each target region of individual samples. The resulting matrices of ratios (fragmentation scores) along with the frequencies of each of the 16 dinucleotides served as predictors for classification.

## Sample classification

The classification was done in R version 4.0.3 within tidymodels framework. The full dataset was split into training and held-out test sets. Data preprocessing included log2-transformation of the density peaks ratios followed by normalization (centering and scaling) of all predictors. Grid search for optimal hyperparameters for the radial basis function support vector machines model (R package kernlab v 0.9-29) was done on the training dataset with 10-times 10-fold cross-validation. The characteristics of the final classifier with the largest area under the ROC curve were estimated on the training set with 10-times 10-fold cross-validation. Ultimately, the final classifier was trained on the full training set and the performance was estimated on the held-out test dataset.

# References

1. Zheng Z, Liebers M, Zhelyazkova B, Cao Y, Panditi D, Lynch KD, et al. Anchored multiplex PCR for targeted next-generation sequencing. Nat Med. 2014;20:1479–84.

2. Martin M. Cutadapt removes adapter sequences from high-throughput sequencing reads. EMBnet.journal. 2011;17:10–2.

3. Langmead B, Salzberg SL. Fast gapped-read alignment with Bowtie 2. Nat Methods. 2012;9:357–9.
