# Supplementary figures and images for: Deep cfDNA fragment end profiling enables cancer detection
Source: Mol Cancer. 2022 Jan 21;21:26. doi: 10.1186/s12943-021-01491-8 (PMC8780681; doi:10.1186/s12943-021-01491-8)

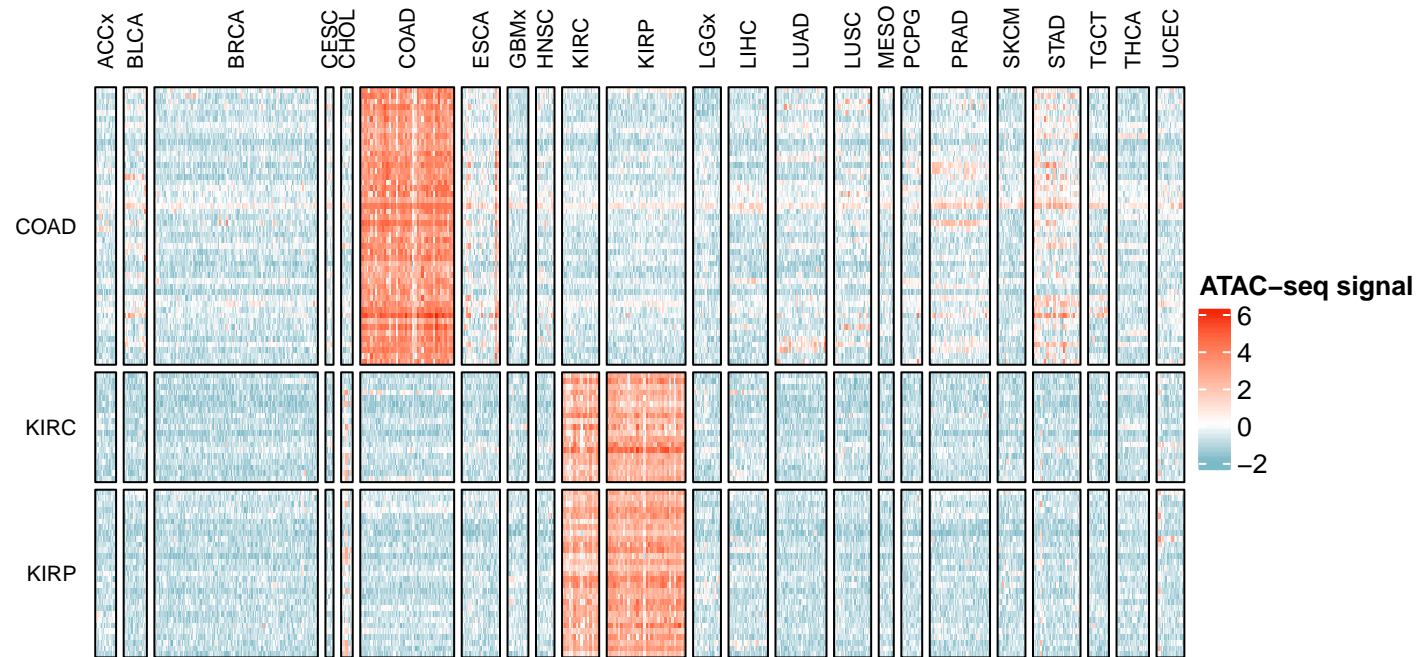

Supplement: Supplementary file 2 — Additional file 2: Supplementary Fig 1. ATAC-seq signal in RCC (KIRP and KIRC) and COAD-specific open-chromatin regions (rows) analyzed in this study shown for the samples (columns) from the TCGA cohort. Data from [10]. [file 12943_2021_1491_MOESM2_ESM.pdf]

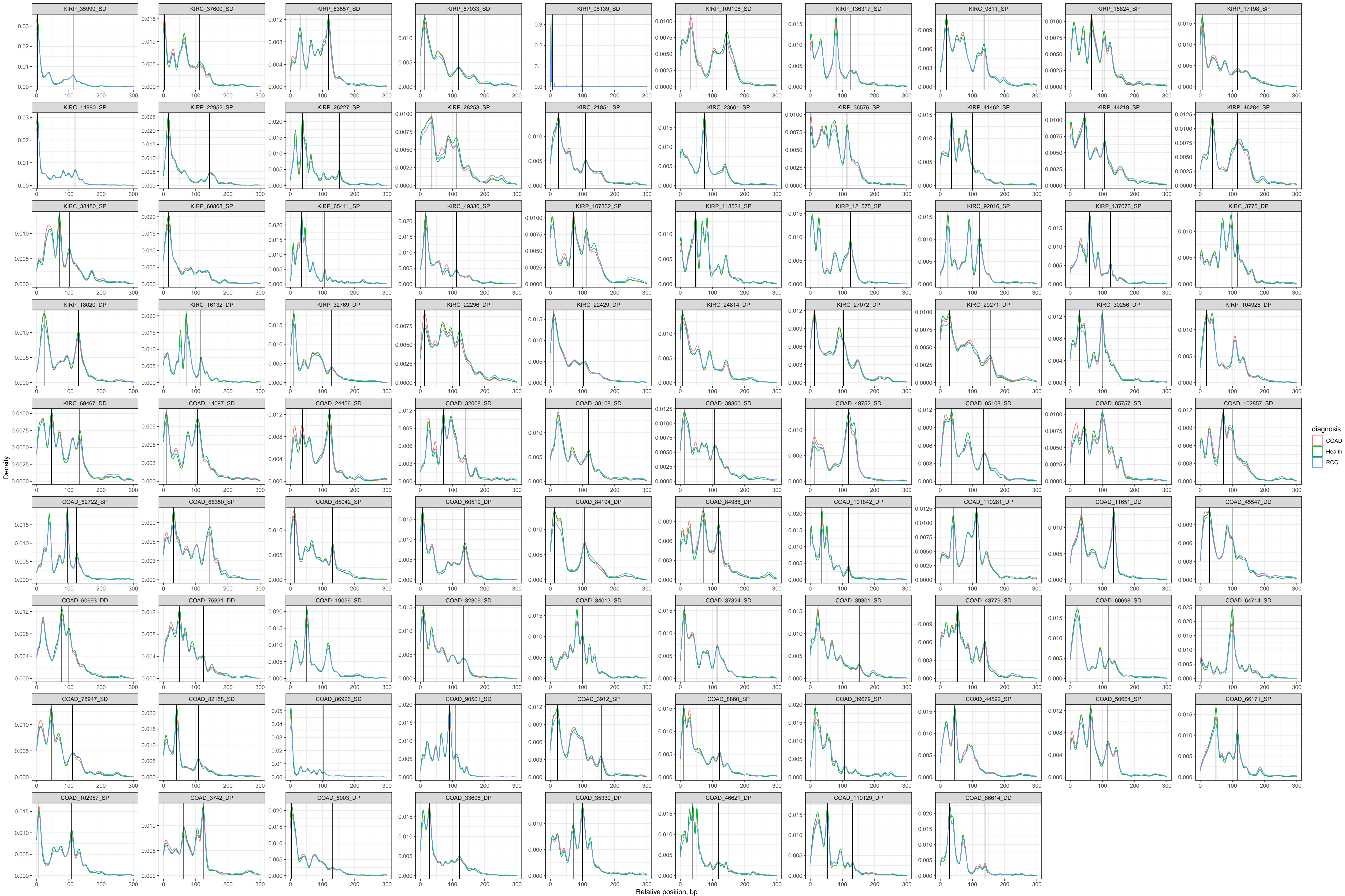

Supplement: Supplementary file 3 — Additional file 3: Supplementary Fig 2. Densities of fragment end distributions in all target regions analyzed in this study plotted for COAD, RCC, and healthy cfDNA samples. Black vertical lines represent positions of Peak1 and Peak2. [file 12943_2021_1491_MOESM3_ESM.pdf]

A

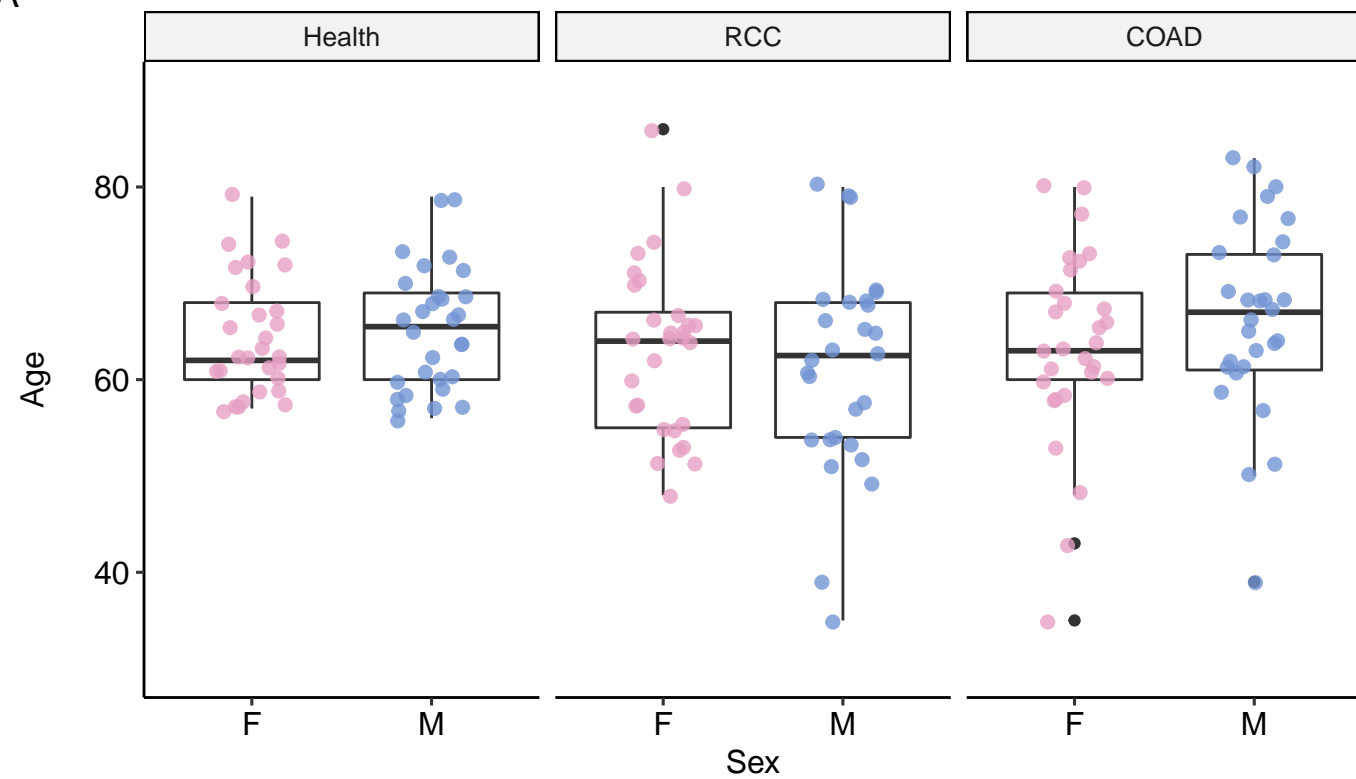

B

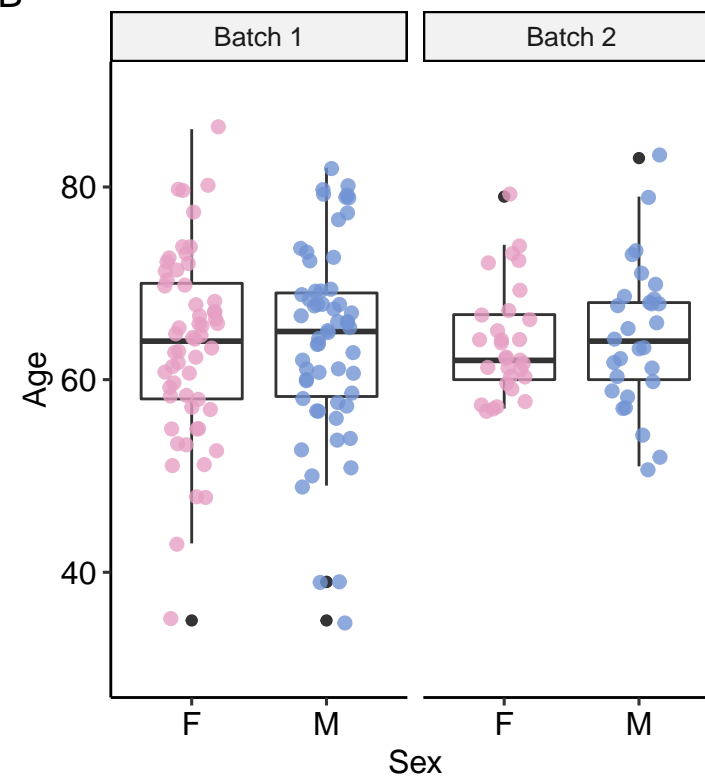

C

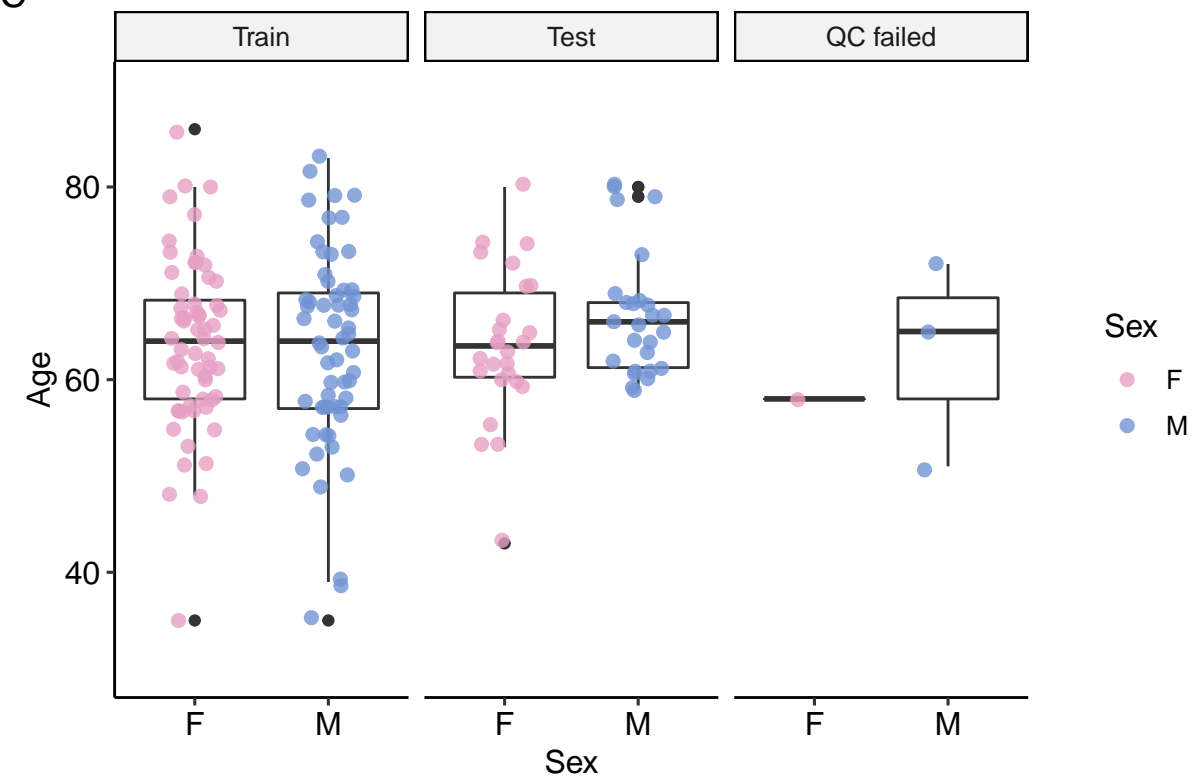

D

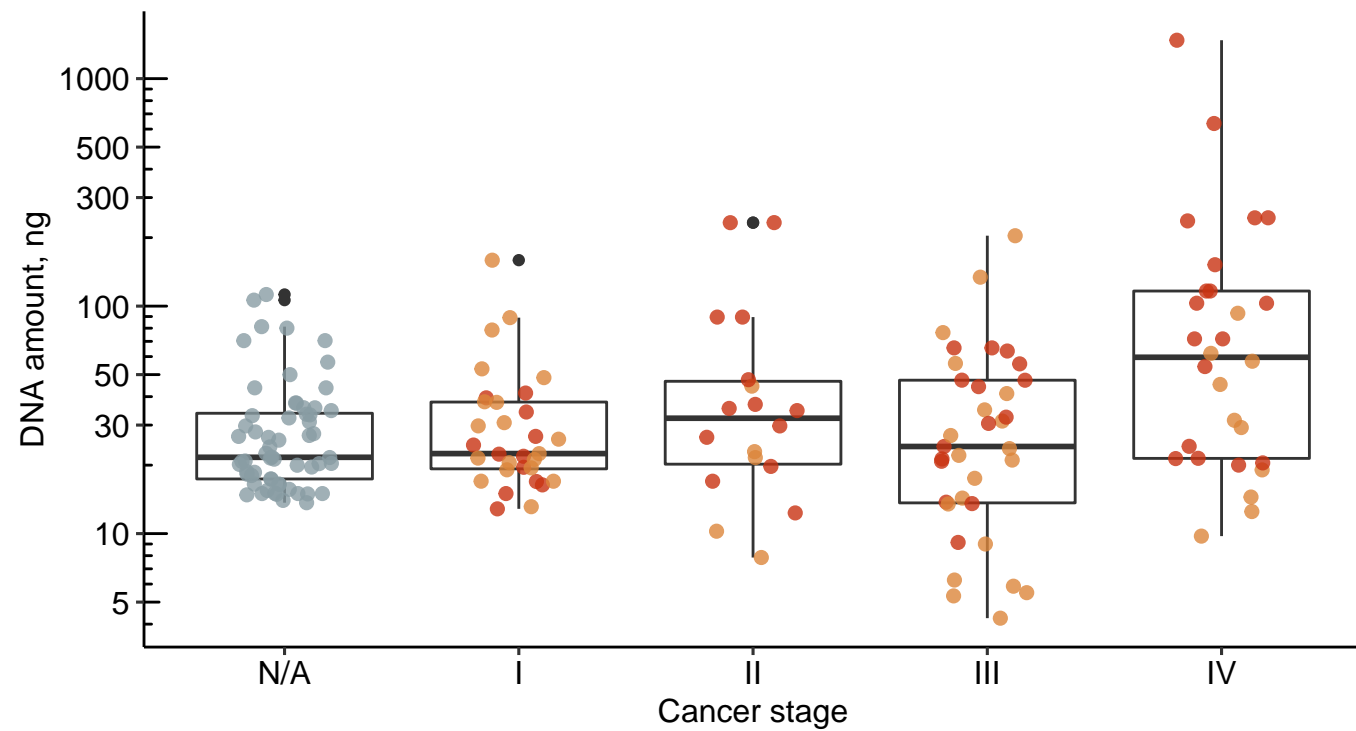

E

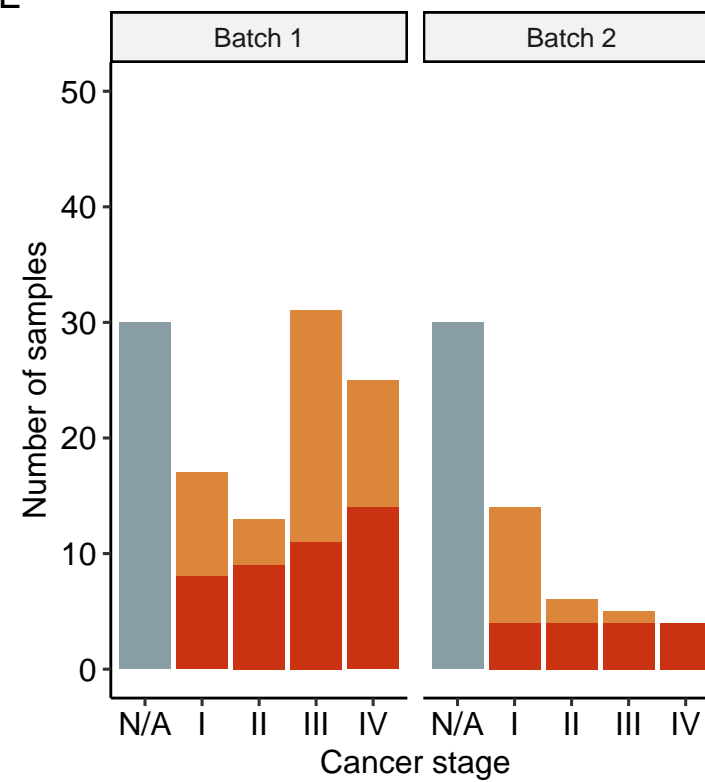

F

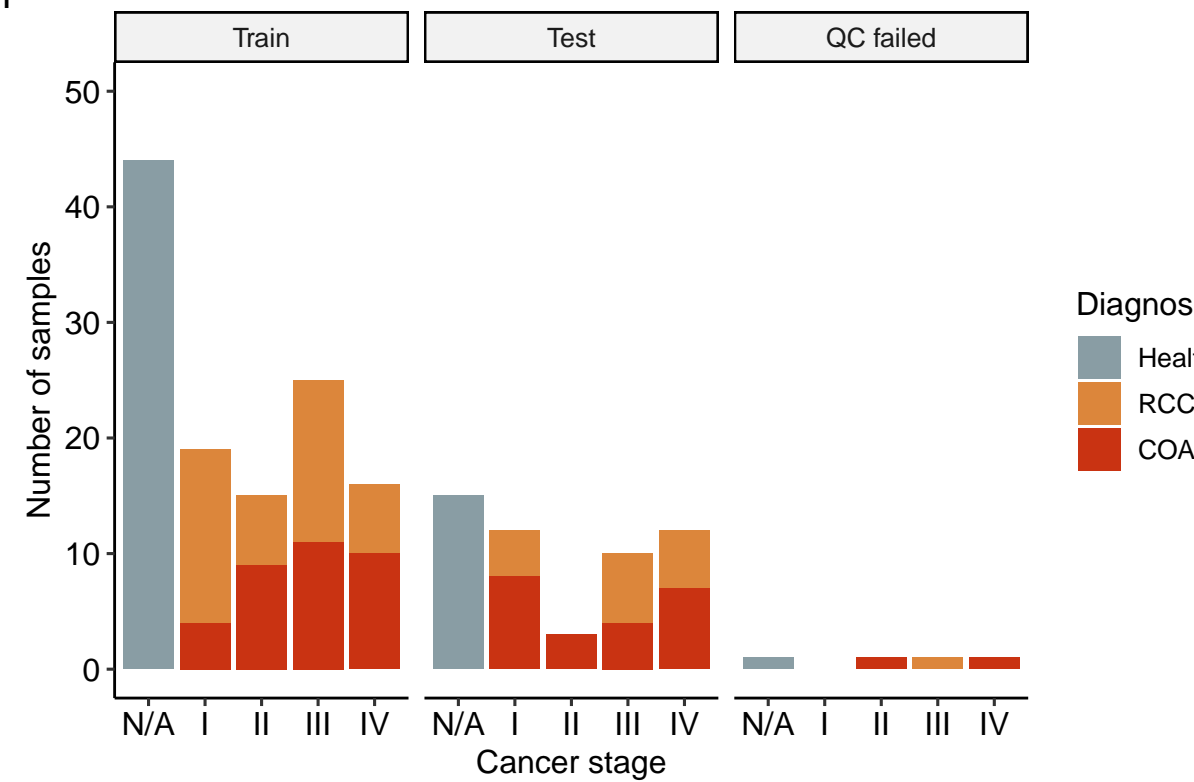

Supplement: Supplementary file 4 — Additional file 4: Supplementary Fig 3. Demographic and clinical characteristics of the cohort. A-C. Age and sex distribution across clinical groups (A), batches (B), and train/test split (C). D. The cfDNA yields across clinical groups and cancer stages. E, F. Stage and diagnosis composition of the batches (E), training and test sets (F). [file 12943_2021_1491_MOESM4_ESM.pdf]
